# Web-based language production experiments: Semantic interference assessment is robust for spoken and typed response modalities

**DOI:** 10.3758/s13428-021-01768-2

**Published:** 2022-04-04

**Authors:** Kirsten Stark, Cornelia van Scherpenberg, Hellmuth Obrig, Rasha Abdel Rahman

**Affiliations:** 1 Humboldt-Universität zu Berlin, Department of Neurocognitive Psychology, 10099 Berlin, Germany; 2 Charité – Universitätsmedizin Berlin, corporate member of Freie Universität Berlin, Humboldt-Universität zu Berlin, and Berlin Institute of Health, Einstein Center for Neurosciences Berlin, Charitéplatz 1, 10117 Berlin, Germany; 3 Humboldt-Universität zu Berlin, Berlin School of Mind and Brain, 10099 Berlin, Germany; 4 Department of Neurology, Max Planck Institute for Human Cognitive and Brain Sciences, Leipzig, Germany; 5 Clinic for Cognitive Neurology, University Hospital and Faculty of Medicine Leipzig, Leipzig, Germany

**Keywords:** Language production, Online experiments, Overt speaking, Keystrokes, Typewritten naming, Cumulative semantic interference, Picture naming

## Abstract

For experimental research on language production, temporal precision and high quality of the recorded audio files are imperative. These requirements are a considerable challenge if language production is to be investigated online. However, online research has huge potential in terms of efficiency, ecological validity and diversity of study populations in psycholinguistic and related research, also beyond the current situation. Here, we supply confirmatory evidence that language production can be investigated online and that reaction time (RT) distributions and error rates are similar in written naming responses (using the keyboard) and typical overt spoken responses. To assess semantic interference effects in both modalities, we performed two pre-registered experiments (*n* = 30 each) in online settings using the participants’ web browsers. A cumulative semantic interference (CSI) paradigm was employed that required naming several exemplars of semantic categories within a seemingly unrelated sequence of objects. RT is expected to increase linearly for each additional exemplar of a category. In Experiment 1, CSI effects in naming times described in lab-based studies were replicated. In Experiment 2, the responses were typed on participants’ computer keyboards, and the first correct key press was used for RT analysis. This novel response assessment yielded a qualitatively similar, very robust CSI effect. Besides technical ease of application, collecting typewritten responses and automatic data preprocessing substantially reduce the work load for language production research. Results of both experiments open new perspectives for research on RT effects in language experiments across a wide range of contexts. JavaScript- and R-based implementations for data collection and processing are available for download.

Conducting experiments online has huge potential to advance behavioural research, beyond the challenges of the current pandemic situation. By running experiments through web browsers and online platforms, large numbers of participants can be recruited for cross-sectional, longitudinal or single-time-point studies at their homes (e.g. Palan & Schitter, 2018). Moreover, access to diverse ethnicities, across countries, age groups and social status is facilitated (e.g. Gallant & Libben, 2019; Peer et al., 2017). Larger and more diverse study populations can increase statistical power and ecological validity (Henrich et al., 2010; Speed et al., 2018). While other fields started to tap into the potential of online experiments more than two decades ago (e.g. Krantz & Reips, 2017), language production experiments—especially when targeting reaction times—have rarely been implemented in web-based settings (but see e.g. Gilquin, 2010 for a non-reaction time language production experiment). This is partially due to the sensitivity to small effects in the range of tens of milliseconds and concerns regarding technical reliability and data quality when measuring overt language production. Recent evidence, however, suggests that studying language production via online platforms is possible. Overt naming responses acquired online have been demonstrated to be precise enough to detect speech onset reaction time effects in the critical range of ~15–50 ms (Fairs & Strijkers, 2021; Vogt et al., 2021). In a picture-naming paradigm, Fairs and Strijkers (2021) replicated lab-based effects of word frequency in an online experiment run on the platform FindingFive (FindingFive Team, 2019) with 100 participants. For the picture word interference (PWI) effect (Bürki et al., 2020; Lupker, 1979), which requires naming object pictures overlaid with semantically related or unrelated distractor words, Vogt et al. (2021) showed online feasibility reproducing the lab-based findings. They implemented the experiment on two different platforms (SoSci Survey, Leiner, 2019 and jsPsych, de Leeuw, 2015) with different participant cohorts (each *n* = 48). A comparison of overt naming and manual name classifications via key press responses revealed similar semantic interference effects for both response modalities, replicating lab-based effects (Abdel Rahman & Aristei, 2010). The results are highly encouraging, but online assessment of overt spoken responses was also shown to require careful planning of the technical setup and a considerable offline processing effort. Specifically, the authors note caveats regarding the large sample size needed for online studies, the increased (technical) noise and the effortful (pre)processing of the data. Similar to lab-based experiments, offline processing of the experimental data, i.e. participants’ audio recordings, requires some cumbersome classification of correctness and post-processing of the vocal onset times within the audio files. There are programs to assist such tasks (Boersma & Weenink, 2020; Roux et al., 2017), but depending on the number of trials and participants, preprocessing the files can still take several days.

Typing instead of overtly pronouncing the word may be an alternative which could ease analyses and application. Indeed, studies have shown that typewritten responses can be a valid alternative modality of language production (Pinet & Nozari, 2018; Torrance et al., 2018). This highlights the fact that the study of written language is coequal to spoken language in many linguistic questions. In an online format, this modality may have much fewer limitations as it can be implemented and processed more easily. Latencies of simple key presses (e.g. “c” for correct) or mouse clicks are regularly used in both lab-based and online experiments. Since their implementation is rather undemanding, they have been implemented in a wide variety of online experiments, including psycholinguistic experiments using categorization tasks (Mathôt & March, 2021; Vogt et al., 2021). Analysing a typed whole word response in language production is slightly more challenging, but it has been shown that typewritten responses can be pre-processed automatically, and a wide range of different procedures exists, even controlling for typing errors (Borrie et al., 2019; Bosker, 2021; Navarro, 2001). Experiments with typewritten answers can thus be an easy-to-implement, time-efficient alternative to spoken responses in reaction time-sensitive language production experiments.

To further explore the potential of web-based experiments targeting language, we here address three questions: (i) Can the well-documented cumulative semantic interference (CSI) effect be replicated in a web-based study design? (ii) How similar is the effect between two modalities, i.e. typed vs microphone-recorded, spoken response? (iii) What recommendations can be provided regarding technical challenges of both approaches?

(i) Can the cumulative semantic interference (CSI) be replicated online?

In Experiment 1, we set out to replicate lab-based language production effects using the CSI paradigm in the same experimental platform and audio-recording method (SoSci Survey; Leiner, 2019; Khan, 2020) as described in Vogt et al. (2021; Experiment 1). The CSI paradigm requires the naming of several exemplars of semantic categories within a seemingly unrelated sequence of objects. In lab-based experiments, reaction times increase linearly for each additional exemplar of a category being named. If this semantic interference effect replicates in an online setting, we confirm the feasibility of time-sensitive overt naming experiments in participants’ web browsers.

(ii) Are web-based recordings of the CSI effect comparable for spoken versus written (typed) response modalities?

In Experiment 2 of the current study, we ran the same language production paradigm on the same experimental platform, but collected typed instead of spoken responses to the target pictures. Since spoken and written language production share underlying linguistic processes, the experiment targets the question whether typing may serve as a reliable alternative response modality in online experiments on language production which are targeting especially timing but also accuracy of the responses.

Of note, writing requires additional skills, acquired later in life. The degree of shared and unique processes in the two response modalities is still a matter of debate, but most theories assume that lexical processing stages are shared across different output modalities (Levelt et al., 1999; Logan & Crump, 2011; Pickering & Garrod, 2013; Roelofs, 2018). Keyboard typing—today undoubtedly the major way of peer-to-peer distant interaction (Brandt, 2015; Pinet, Dubarry, & Alario, 2016a)—and handwriting diverge regarding certain aspects of motricity and motor planning (e.g. for complex writing systems Higashiyama et al., 2015), but research suggests that they share central linguistic mechanisms (e.g. Pinet, Ziegler, & Alario, 2016b; see also Qu et al., 2020). With regard to written versus spoken responses, it has been shown that both are modulated by lexical frequency, age of acquisition and image agreement (Bertram et al., 2015; Bonin et al., 2002; Pinet, Ziegler, & Alario, 2016b; Torrance et al., 2018). Moreover, phonological priming effects are similar in both modalities (Breining et al., 2016; Chen & Li, 2011; Qu & Damian, 2020; Roux & Bonin, 2012; Zhang & Damian, 2010). For picture naming, written naming was mostly found to be slower compared to spoken naming (Bonin & Fayol, 2000; Chen & Li, 2011). Interestingly, differences tend to disappear when participants see what they write (Perret & Laganaro, 2013; Snyder et al., 2015). Extending the modality comparison to web-based assessment may be of special interest in future studies targeting cohorts with special requirements since they may profit from assessing the effect in one rather than the other modality (e.g. people with aphasia, people with dysarthria).

(iii) What are the technical challenges and how can we address them?

Regardless of response modality, technical demands of online experiments involve specific computational and hardware/software-related characteristics (see e.g. Grootswagers, 2020 for an overview of the general infrastructure of online experiments). One computational aspect is the integration of recording of the audio input and the typing latencies themselves. Recent JavaScript-based implementations make this possible. JavaScript is a programming language native to all modern browsers and thereby does not need installation prior to testing, either on the programmers’ or on the users’ side. In combination with HTML and CSS, it forms the core technology of the World Wide Web. It is event-driven; that is, it allows for programming reactions to any “event” with high temporal precision and without reloading the web page. Events can, for instance, be key presses or mouse clicks. JavaScript can also time the presentation of elements, such as pictures and texts, defined and styled with HTML and CSS. JavaScript-based implementations can be combined with most platforms for online surveys and experiments which allow the experimenter to enter JavaScript code chunks. For the current study, audio recordings were acquired using a JavaScript plugin available on GitHub (Khan, 2020). For detecting key stroke latencies, we programmed a custom JavaScript (Stark, 2021b) which we make available for download.

Regarding hardware and software demands, a major aspect is the variability between participants. A precise time lock between stimulus presentation and onset of the recording or timer is crucial. In the lab, the technical properties can be controlled and are mostly stable across participants. In online experiments, the hardware and software varies between participants and can corrupt data quality and signal-to-noise ratio (Anwyl-Irvine, Dalmaijer, et al., 2020a; Bridges et al., 2020). Sources of variance are the experimental platform and browser used, the operating system and the type and quality of participants’ microphones and their interface to the further hardware. For instance, one study found that the interface between audio system and operating computer (analogue-to-digital and digital-to-analogue conversion) introduced uncontrolled latency jittering of about 5–10 ms (Kim et al., 2020). However, when put into practice, the overall noise seems to affect data quality less than expected. Being relatively stable for a single participant, we can assume relatively high precision for within-subject comparisons with a sufficient amount of trials (Bridges et al., 2020; Pinet et al., 2017; Vogt et al., 2021; see Baker et al., 2020 for a recent article on how sample size and number of trials affect statistical power). Regarding key presses, a previous study compared the objective timing of different devices (Reimers & Stewart, 2015). Absolute overestimation of RTs ranged from 30 to 100 ms on different hardware/software systems and web browsers. The variability within a single system, however, was comparatively low (< 10 ms). Hence, the authors conclude that “within-subject comparison of response times across two conditions is almost unaffected” in web-based research. Although estimations of the actual noise introduced by a single participants’ system are extremely difficult regarding both within- and between-participant data, the reported results are encouraging (see also more recent studies by Anwyl-Irvine et al., 2020a, b; Bridges et al., 2020). We thus proceed from the assumption that the additional noise does not affect the interpretation of within-subject comparisons if the effects are well above 10 ms and if no randomization between participants is necessary.

In summary, the two experiments presented here are aimed at (i) providing confirmatory evidence that web-based language production experiments yield reliable within-subject effects if a sufficient number of trials is employed (Experiment 1). Furthermore, we investigated (ii) whether reaction time (and, exploratory, error rate) effects are comparable for typewritten and spoken response modalities (Experiment 2). Addressing the issue of preprocessing after data collection, we compared manual and automatic classification procedures of typewritten answers. This aimed at (iii) improving the workflow and technical ease of application. To encourage broader use of web-based language production research, we provide materials and guidelines that may help researchers to plan their own reaction time experiments online.

Both experiments (Experiment 1: spoken responses; Experiment 2: typed responses) were programmed and run on the platform SoSci Survey (Leiner, 2019), a Germany-based platform for conducting social and behavioural research. For Experiment 1, audio recording was implemented using the JavaScript-based plugin RecordRTC (Khan, 2020). For Experiment 2, the keystroke onsets were detected using customized JavaScript (Stark, 2021b). Materials, design and procedure of the two experiments were largely identical and are described in detail below and visualized in Fig. 1. Both experiments and the analyses were preregistered on the open science framework (Experiment 1: https://osf.io/dbmpu; Experiment 2: https://osf.io/s5gy3).

**Fig. 1:**
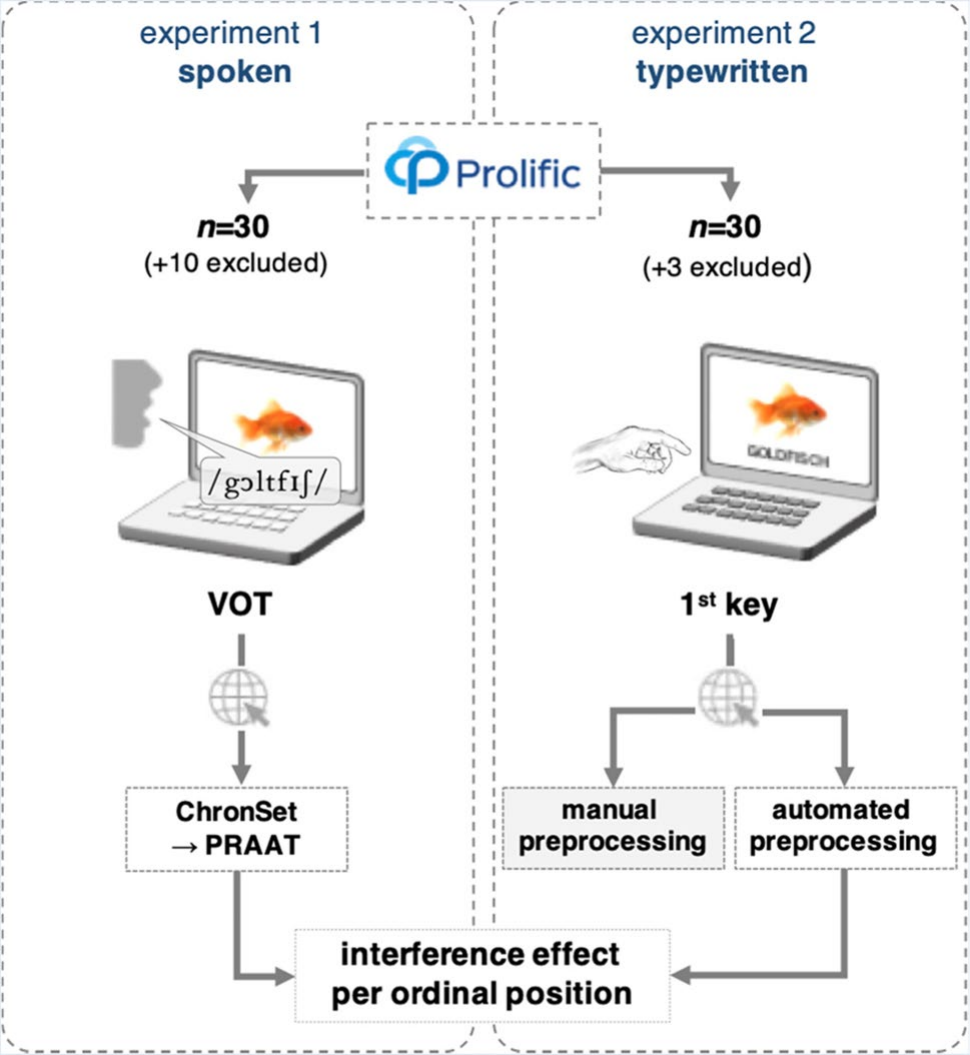
Overview of the experimental procedures in Experiments 1 and 2

## Experiment 1: CSI with Spoken Responses

### Methods

#### Participants

Forty native German speakers between 18 and 35 years of age were recruited via the commercial platform Prolific (www.prolific.co.uk) and completed the full experiment. Following the preregistered criteria, the final sample comprised only participants who reached a minimum of 80% of valid or correct trials and passed the criteria to ensure sufficient attention during online performance. Accordingly, we excluded one participant who had failed the second attention check (item vs non-item), one due to missing audio recordings, and eight due to too many null responses or other errors resulting in trial loss >80%. To determine the necessary final sample size, we ran an a priori power analysis using the R package simr (Green & MacLeod, 2016) based on estimates from a previous, lab-based continuous naming study (Rose & Abdel Rahman, 2016). This resulted in a suggested sample size of 24 for a power estimate of 80%. To account for expected noise in the data sample due to the online setting, we a priori decided to increase the estimated sample size by 25%. The final sample thus consisted of 30 participants (16 female, aged 19–35, *M*_age_ = 26.5, *SD*_age_ = 5.1).

Experimental procedures were approved by the Institutional Review Board of the University of Leipzig, Germany, in accordance with the Declaration of Helsinki (amendment to ethical approval AZ 144/18-ek, Ethics Committee University Leipzig). All participants gave their informed consent at the beginning of the study and were rewarded monetarily.

#### Material

The 160 experimental stimuli used in the study were coloured photographs of everyday objects. The 120 target stimuli consisted of 24 semantic subcategories with five closely related members each. For example, /shark/, /eel/, /ray/, /goldfish/ and /dolphin/ constituted the subcategory “fish” as part of the superordinate category “animals”. Other categories included fruit (food), seating furniture (furniture); for a full list see Appendix Table 8. Additionally, 40 fillers were added to the overall item set (25%).

#### Design

The 24 categories were distributed across eight blocks of three categories each. To each block, five filler items were added, resulting in eight blocks of 20 items. Five block orders were created in a pseudorandomized fashion such that categories that shared a superordinate semantic category (e.g. fish and insects: animals) were as far apart as possible. Trial randomization was done using the program MIX (Van Casteren & Davis, 2006). Six randomized trial lists were created for each block order, resulting in a total of 30 randomized lists. When participants opened the survey in Prolific but did not complete the experiment, they were assigned a randomized list but were not listed amongst the 40 paid participants. Due to the random assignment of the lists by SoSci Survey, few lists were hence used several times, whereas others were not used at all. Trial randomization was constrained in that within each block, members of each category were separated by at least two (lag = 2) and a maximum of eight items (lag = 8), including fillers and members of different categories. Note that previous research suggests that *lag*, i.e. the distance between two ordinal positions within one semantic subcategory, does not affect the cumulative semantic interference effect across ordinal positions (e.g. Schnur, 2014).

#### Procedure

The experiment started with an instruction of the general procedure to which the participants consented. They were then familiarized with the materials by presenting eight pictures each on the screen with their names written underneath. The participants were instructed to look at the pictures closely, read their names (if possible, aloud), and to proceed to the next set of pictures in a self-paced manner by pressing the space bar or enter key. After familiarization, a catch trial showed two previously seen and two novel items to check whether participants had paid attention to the pictures. Response was mandatory but was only used for later assessment of data quality, and participants were able to proceed regardless of their answer. This was followed by instructions to allow the browser to access the computer microphone. After that, participants were instructed to name each presented picture as quickly and accurately as possible. Following four practice trials, the main task started. After a fixation cross, presented for 500 ms, the target picture appeared for 2 s. The audio recording was started with the appearance of the picture and lasted for 2.5 s. The next trial started automatically. After completion of the 160 trials, the experiment finished with another attention check (two previously named and two novel items), a debriefing page and the option to leave comments. The whole experiment lasted around 15 min on average (range = 10–20 min).

#### Data Processing

The recorded audio files were retrieved from the SoSci Survey server and converted into wav files. Vocal onset times (VOTs) were detected using the Chronset algorithm (Roux et al., 2017) and checked manually using a customized Praat script (Boersma & Weenink, 2020; van Scherpenberg et al., 2020). The final VOTs were determined at the start of each word, excluding stuttering or “uhms”. These VOTs were considered the overt response, i.e. the reaction times.

From the overall 4800 observations (160 trials × 30 participants), 100 were excluded due to missing responses or technical errors. A total of 269 trials were excluded due to incorrect naming. These included semantic errors (e.g. “car” for “carriage”, *n =* 187), naming of unrelated words (e.g. mushroom for ball, *n =* 31) or other errors such as naming of articles before the word or stuttering (*n* = 51). On average, 7.69% of participants’ responses were considered as incorrect (*SD =* 3.85%).

Statistical analyses were thus based on 3264 observations (4800 observations excluding the 1200 filler trials (40 trials × 30 participants) and 369 erroneous trials).

### Statistical Analyses

Statistical analyses for both experiments were done in R (version 4.0.2; R Core Team, 2020). Following the procedure suggested by Lo and Andrews (2015), to account for the non-normal, skewed distribution of the raw reaction time data, generalized linear mixed models (GLMM) were run with a gamma distribution and identity link function using the R package lme4 (version 1.1-23; Bates et al., 2014). *P*-values were calculated using the Wald *Z-*statistics. Reaction times (RTs) were entered as the dependent variable, and ordinal position as a continuous, mean-centred predictor. The model converged with the fully specified crossed random effects structure (Barr et al., 2013) including intercepts and slopes of the ordinal position for both subjects and categories. For the exploratory analyses of error rates, a generalized linear mixed model with a binomial distribution was computed. Initially, the model was specified with the same fully crossed random effects structure as for the RT analyses. Due to convergence problems, we then adopted our preregistered model reduction procedure, following the recommendations by Brauer and Curtin (2018) by increasing the number of optimizer iterations to 2 × 10^5^, and subsequently reducing the random structure. The model converged with a random intercept for subjects and a random intercept and slope for categories. Anonymized data and scripts are provided on the Open Science Framework: https://osf.io/w6ptm/.

### Results

**Fig. 2:**
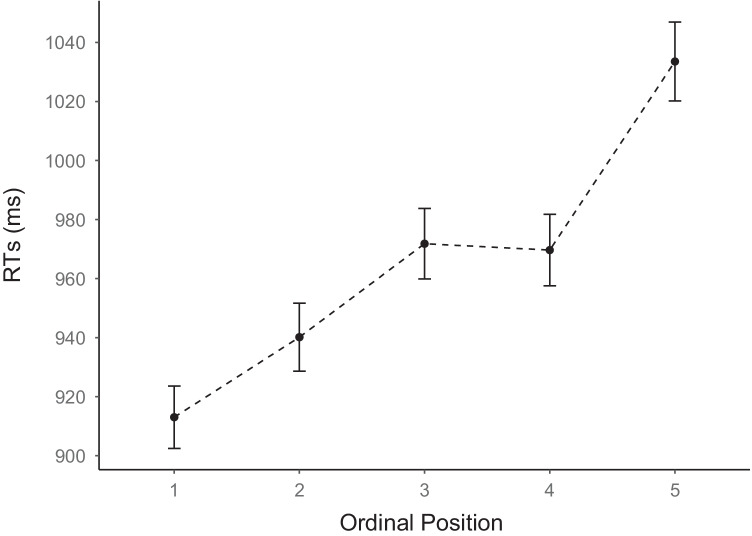
Mean naming latencies (RTs) in milliseconds as a function of ordinal position. *Note.* Mean reaction times (RTs) were calculated across semantic categories and participants. Error bars show standard errors of the mean. Values were adjusted for within-participant designs using the method suggested by Morey (2008) as implemented in the *summarySEwithin(* ) function from the R package Rmisc (Hope, 2013)

The mean RTs across ordinal positions are visualized in Fig. 2. As can be seen, they follow a linear increase with a plateau at ordinal position 4. To confirm this linear trend statistically, we ran a GLMM as described above with a fully specified random structure. The results are summarized in Table 1. This confirmed that RTs increased significantly with an average of ~31 ms per ordinal position. As shown in Table 2, error rates also increased with ordinal position. Averages for each participant and each category (RTs and error rates) are provided in Appendix Figs 6 and 7.

**Table 1:** Generalized linear mixed model (GLMM) with gamma identity link function predicting vocal onset latencies (RTs) by ordinal position

Effect	Estimate	*SE*	95% CI		*t-*value	*p*
			*LL*	*UL*		
Model: RT ~ ordinal position + (ordinal position | subject) + (ordinal position | category)						
Fixed effects						
Intercept	1007.04	7.78	991.80	1022.28	129.51	**< .001**
Ordinal position	30.78	5.87	19.29	42.28	5.25	**< .001**

Number of participants = 30; number of categories = 24; total *N =* 3264; *SE* = standard error; *CI =* confidence interval around the estimate; *LL =* lower limit; *UL =* upper limit. *P*-values are based on a Wald *Z*-test. Significant *p*-values of *p* < .05 are shown in bold

**Table 2:** GLMM with binomial distribution predicting error rates by ordinal position

Effect	Log-odds	*SE*	95% CI		*z-*value	*p*
			*LL*	*UL*		
Model: errors ~ ordinal position + (1 | subject) + (ordinal position | category)						
Fixed effects						
Intercept	−2.74	0.24	−3.20	−2.27	−11.45	**< .001**
Ordinal position	0.15	0.06	0.04	0.26	2.64	**.008**
Percentage of erroneous trials	Ordinal position					
	1	2	3	4	5	
* M*	6.94	9.58	8.06	9.72	12.36	
* SEM*	0.85	0.85	0.88	1.11	1.07	

Number of participants = 30; number of categories = 24; total *N =* 3600; *SE* = standard error; *CI =* confidence interval around the estimate; *LL =* lower limit; *UL =* upper limit; *M =* mean; *SEM =* standard error of the mean (Morey, 2008); erroneous trials *=* number of trials per ordinal position that were excluded due to errors (technical or answer-based). *P*-values are based on a Wald *Z*-test. Significant *p*-values of *p* < .05 are shown in bold

## Experiment 2: CSI with Typewritten Picture Naming

### Methods

#### Participants

For Experiment 2, another group of 33 native German speakers aged between 18 and 35 years was recruited via Prolific (www.prolific.co.uk), none of whom had participated in the first experiment. All participants entered the experiment using a computer or laptop and a QWERTZ keyboard, the most widely used keyboard type in Germany. Based on our preregistered inclusion criteria, which were identical to Experiment 1 (a minimum of 80% of valid trials in the CSI task and correct answers in the attention checks), three participants were excluded^1^. Thus, the final sample consisted of 30 participants (nine female; four left-handed; aged 18–35, *M*_age_ = 25.4, *SD*_age_ = 4.6). Their mean typing speed was 15.3 five-character words per minute (*SD =* 7.5; range 4.0 to 34.1), and their accuracy was 80% (*SD =* 9.5; range 64 to 94), as assessed by a typing test (see below).

Sample size was determined to be identical to Experiment 1, and an a priori power analysis (simr, version 1.0.5; Green & MacLeod, 2016) based on the fixed and random estimates from Experiment 1 suggested a power >85% to detect an effect of similar magnitude with 30 participants. Experimental procedures were approved by the local ethics board of the Humboldt-Universität zu Berlin, in accordance with the Declaration of Helsinki (ethical approval 2020-68). All participants gave their informed consent and were rewarded monetarily.

### Material, Design and Procedure

The 160 experimental stimuli were identical to Experiment 1, and design and procedure of both experiments were kept largely identical. The different response modality resulted in four modifications of the design of Experiment 1: (1) Upon opening the study link, participants were screened for using a QWERTZ keyboard (i.e. the six first letters of the upper letter row being Q, W, E, R, T and Z) using a custom JavaScript plugin (based on the comparison of the *event.key( )* and *event.code( )* methods, available at https://github.com/kirstenstark/typing_RTs_JS). This was done to ensure that all participants had direct keys for all German letters (e.g. “ö” and “ß”) and that key positions were identical between participants. (2) During the familiarization with the material, pictures and picture names were presented one after another at a central position on the screen. For each picture, the participants were instructed to type the picture name at their own pace in a text box displayed underneath the picture. Participants saw what they typed and were allowed to correct answers using the backspace button. To keep the familiarization comparable to Experiment 1, no feedback on the correctness of the typed answer was given. Prior to the familiarization, participants had been instructed to enable the caps lock key and to write all letters in upper case. Regardless of whether they adhered to the instructions, the typed characters were always displayed in capital letters. Because German nouns start with a capital letter, this was done to accustom participants to not press the shift key upon the beginning of each typed word. (3) During the main experimental task, participants were instructed to type the name of each presented picture as quickly and accurately as possible. They were informed that single spelling mistakes (“typos”) were not a problem. In each trial, a fixation cross—identical to Experiment 1—was presented for 500 ms after the page was fully loaded. The following target picture was displayed for 6 s (as opposed to 2 s in Experiment 1) or until the space bar or enter key was pressed. Like during familiarization, the typed answers appeared in a text box below the target picture, and corrections using the backspace key were allowed. (4) At the end of the experiment, to achieve an accurate sample description, participants performed a typing test in which they copied three texts of ~155 characters each in their usual typing speed. Participants’ typing accuracy and speed were calculated by taking the percentage of five-character words containing no errors or backspaces and by dividing the number of correct five-character words by the total time needed for all five-character words (see Crump & Logan, 2010; Pinet, Dubarry, & Alario, 2016a). The whole experiment lasted around 28 min on average (range = 19–55 min).

Although the experimental platform SoSci Survey is mainly PHP-based, HTML, CSS and JavaScript code can be implemented to customize a survey. Thus, keystrokes, keystroke latencies and typed words were collected in the main experimental task using a custom script that relied on the JavaScript *document.addEventListener( )* and *keydown( )* methods, and the general JavaScript object *Date().* The JavaScript code and an implementation for SoSci Survey are available on GitHub (Stark, 2021b).

#### Data Processing

##### Manual Preprocessing

The correctness of collected word entries was classified half automatically based on our preregistered trial exclusion criteria, using custom scripts in R and Excel. Word entries were considered as correct if the expected picture name or an accepted synonym (see Appendix Table 8) was entered. As reaction time analyses relied on the latency of the first keystroke only, word entries were also considered correct when the first character was correct and the typed word was recognizable for the coding experimenter despite typing errors. For each valid trial, the latency of the first keystroke was considered as the beginning of the overt response, i.e. the reaction time.

##### Automated Preprocessing

In comparison to the time-consuming preprocessing of spoken responses, the preprocessing of typed responses is less effortful because reaction times can be determined online. However, manually classifying the correctness of typed word entries still takes a considerable amount of time (Borrie et al., 2019). Automated assessment of typed responses can be a highly efficient and replicable method (within and between raters) to further reduce the effort (Bosker, 2021). To test the applicability of automated assessment in typed picture naming, we compared our semi-automatic/manual classification to an automated classification procedure using the Jaro distance. The Jaro distance (Jaro, 1989, 1995) is a heuristic metric that compares character strings based on the number of and distance between matching characters, assuming that mismatches and transpositions between close characters are more likely to represent typing mistakes than mismatches between distant characters. It is implemented in the *stringdist(method = “jw”, p = 0)* function of the stringdist package in R (version 0.9.6.3; van der Loo, 2014). The metric is bounded between 0 and 1 (0 representing identical strings and 1 representing complete dissimilarity) and tailored specifically to human-typed, rather short strings (Bosker, 2021; van der Loo, 2014).^2^ For the exact formula applied, we may refer to van der Loo (2014).

During the automated preprocessing, we (1) deleted space or enter keys at the end of a word string, (2) computed backspace-corrected word entries, e.g. by replacing “CHEBackspaceAIR” with “CHAIR”, and (3) calculated the Jaro distance *d*_Jaro_ between each backspace-corrected word entry and the picture name or accepted naming alternatives. The list of accepted naming alternatives was generated before and during the manual classification of spoken (Experiment 1) and typed responses (Experiment 2). A “best match” alternative naming was favoured over the actual picture name when the first character of the typed word entry and the alternative were identical and their Jaro distance was lower than the distance between word entry and picture name. (4) Finally, word entries were classified for correctness and different error types. A word entry was classified as correct if the first letter was typed correctly (i.e. item or “best match” alternative and word entry started with the same character, before and after backspace correction) and the Jaro distance was *d*_Jaro_ < .3. A word entry was classified as incorrect if the first typed key was a special character, such as shift, space or backspace, or an incorrect character, or if the Jaro distance was *d*_Jaro_ ≥ .3. All steps described above were implemented in separate R functions which can be found on GitHub (Stark, 2021a; https://github.com/kirstenstark/stringmatch_typed_naming).

### Statistical Analyses

#### Manual vs Automated Preprocessing

We compared the manual/half-automatic and automated classification procedures and found that, across participants, only 0.60% of all trials were classified differently: Of the 4800 trials, eight trials manually classified as incorrect were classified as correct in the automated procedure (“new correct trials”), and 21 trials manually classified as correct were now considered as incorrect (“new incorrect trials”). The classification differences mainly occurred for the following reasons: (1) Participants backspace-corrected an accepted alternative, changing the first character of the word entry (*n* = 13 new incorrect words; e.g. BURBackspaceBackspaceBackspaceBackspaceSCHLOSS [BUR(G) vs SCHLOSS; engl. *castl(e)…fortress*]), (2) they misspelled the beginning of a word with a phonologically similar phoneme (*n =* 6 new incorrect words; e.g. PFEILE instead of FEILE [engl. similar to *wrasp* instead of *rasp*]), (3) they typed orthographically similar words (*n =* 2 new correct words; e.g. KESSEL [engl. *kettle*] instead of KELLE [engl. *ladle*]) or (4) they typed only parts of the picture name (*n =* 5 new correct words; e.g. GESCHIRR [engl. *dish*] instead of GESCHIRRSPÜLER [engl. *dishwasher*]). These unwarranted misclassifications can be considered negligible since the correlation between manual/semi-automatic and automated classification was close to perfect (Pearson’s *r =* .97). The formula-based automated classification matched the intuitive manual classification almost completely. Therefore, we hereafter report the results based on the automated classification procedure. For comparison, we report the RT results based on the manual classification in Appendix 4, which are largely identical.

#### Typing Errors

On average, 10.85% (*SD =* 4.73%) of word entries per participant were classified as incorrect. The different error types are summarized in Table 3. The final statistical analyses of reaction times were thus based on 3178 observations (4800 observations excluding the 1200 filler and 521 erroneous trials [99 erroneous trials were fillers]), while the exploratory analyses of error rates were based on all 3600 observations.

**Table 3:** Automated classification of typed word entries

Correct	Total	Based on picture name			Based on alternative naming		
		Identical	Corrected	*d*_Jaro_ < .3	Identical	Corrected	*d*_Jaro_ < .3
	4279 (89%)	3519 (82.24%)	301 (7.03%)	133 (3.11%)	-	302 (7.06%)	24 (0.56%)
Incorrect	Total	*NA*	Special key start	Shift start	*d*_Jaro_ ≥ .3	First letter error	Combined
	521 (11%)	153 (29.37%)	25 (4.80%)	10 (1.92%)	12 (2.30%)	231 (44.34%)	90 (17.27%)

Identical = participants typed the exact (alternative) picture name; corrected = participants backspace-corrected their word entry to the exact (alternative) picture name; *d*_Jaro_ < .3 = the Jaro distance between participants’ backspace-corrected word entries and (alternative) picture name was below .3; *NA =* no keystroke was detected (no answer given or technical error); special key start = participants started by pressing the space, backspace, caps lock or enter key; shift start = participants started by pressing the shift key (which would be correct for German nouns if participants had not been instructed to enable the caps lock key and write everything in upper case); *d*_Jaro_ ≥ .3 = the Jaro distance between participants’ backspace-corrected word entries and (alternative) picture names was greater than or equal to .3; first letter error = the first typed characters of word or backspace-corrected word were different from the first letter of the (alternative) picture name; combined = the Jaro distance exceeded the threshold for correctness (*d*_Jaro_ ≥ .3) and the first typed letter was incorrect (first letter error)

To analyse participants’ errors of typed responses, a generalized linear mixed model (GLMM) with a binomial distribution was fitted to predict the error rates by ordinal position. The model converged after increasing the number of iterations to 2 × 10^5^ and restricting the correlation parameters to zero. *P*-values were calculated using the Wald *Z-*statistics.

#### Reaction Time Analyses

As for Experiment 1, the distribution of the raw reaction times was skewed. Therefore, a GLMM with a gamma distribution and identity link function was fitted to predict reaction times (RTs) by continuous, mean-centred ordinal position. *P*-values were calculated using the Wald *Z-*statistics. The model converged with the fully specified crossed random structure (Barr et al., 2013) including intercepts and slopes of the ordinal position for both subjects and categories. Anonymized data and scripts can be found on the open science framework: https://osf.io/gmnc8/.

### Results

**Fig. 3:**
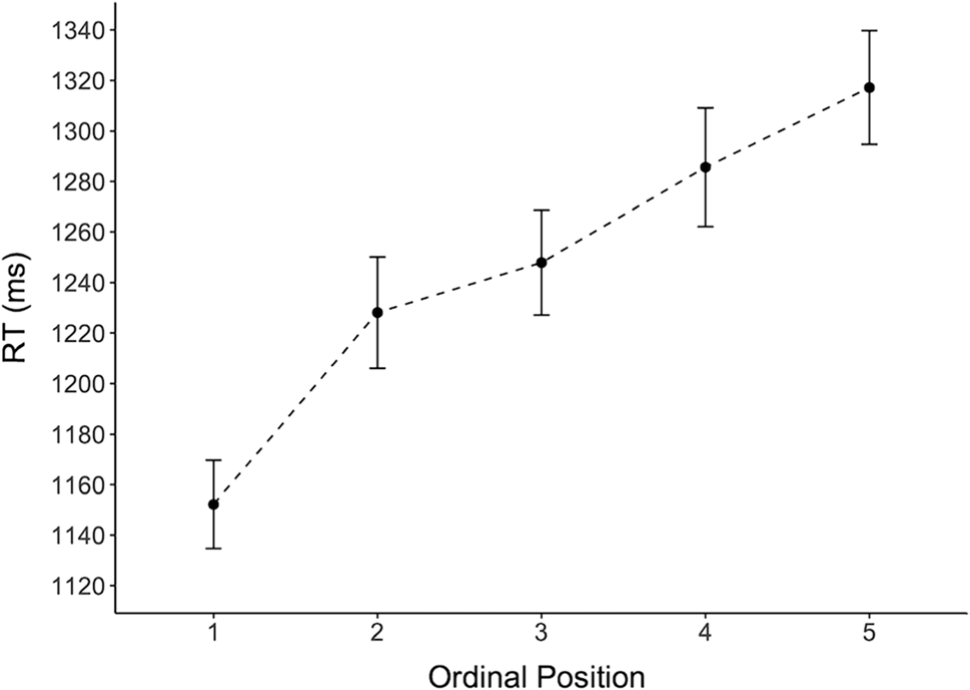
Mean typing latencies (RTs) in milliseconds as a function of ordinal position. *Note.* Mean reaction times (first keystrokes) were calculated across semantic categories and participants. Error bars show standard errors of the mean. Values were adjusted for within-participant designs using the method suggested by Morey (2008) as implemented in the *summarySEwithin(* ) function from the R package Rmisc (Hope, 2013)

The mean reaction times, i.e. the latencies between picture onset and first keystroke (visualized in Fig. 3) show a linear increase with ordinal position. The GLMM confirmed this linear trend: RTs increased significantly with an average of ~42 ms per additional member of each category (Table 4). The error rates did not differ statistically between ordinal positions (Table 5). See Appendix Figs. 8 and 9 for a visualization of the CSI effect and of the error rates for each participant and category separately.

**Table 4:** Generalized linear mixed model (GLMM) with gamma identity link function predicting typing latencies (RTs) by ordinal position

Effect	Estimate	*SE*	95% CI		*t*-value	*p*
			*LL*	*UL*		
Model: RT ~ ordinal position + (ordinal position | subject) + (ordinal position | category)						
Fixed effects						
Intercept	1298.49	11.43	1276.08	1320.90	113.56	**< .001**
Ordinal position	41.68	6.83	28.29	55.06	6.10	**< .001**

Number of participants = 30; number of categories = 24; total *N =* 3178; *SE* = standard error; *CI =* confidence interval around the estimate; *LL =* lower limit; *UL =* upper limit. *P*-values are based on a Wald *Z*-test. Significant *p*-values of *p* < .05 are shown in bold. The model structure was identical to Experiment 1 (see Table 1)

**Table 5:** GLMM with binomial distribution predicting error rates by ordinal position

Effect	Log-odds	*SE*	95% CI		*z-*value	*p*
			*LL*	*UL*		
Model: Errors ~ ordinal position + (ordinal position || subject) + (ordinal position || category)						
Fixed effects						
Intercept	–2.25	0.16	–2.57	–1.93	–13.89	**< .001**
Ordinal position	0.05	0.05	–0.04	0.15	1.06	.291
Percentage of erroneous trials	Ordinal position					
	1	2	3	4	5	
* M*	10.42	12.08	11.39	11.53	13.19	
* SEM*	1.34	1.17	1.35	1.26	1.77	

Number of participants = 30; number of categories = 24; total *N =* 3600; *SE* = standard error; *CI =* confidence interval around the estimate; *LL =* lower limit; *UL =* upper limit; *M =* mean; *SEM =* standard error of the mean (Morey, 2008); erroneous trials *=* number of trials per ordinal position that were excluded due to errors (technical or answer-based). *P*-values are based on a Wald *Z*-test. Significant *p*-values of *p* < .05 are shown in bold

**Fig. 4:**
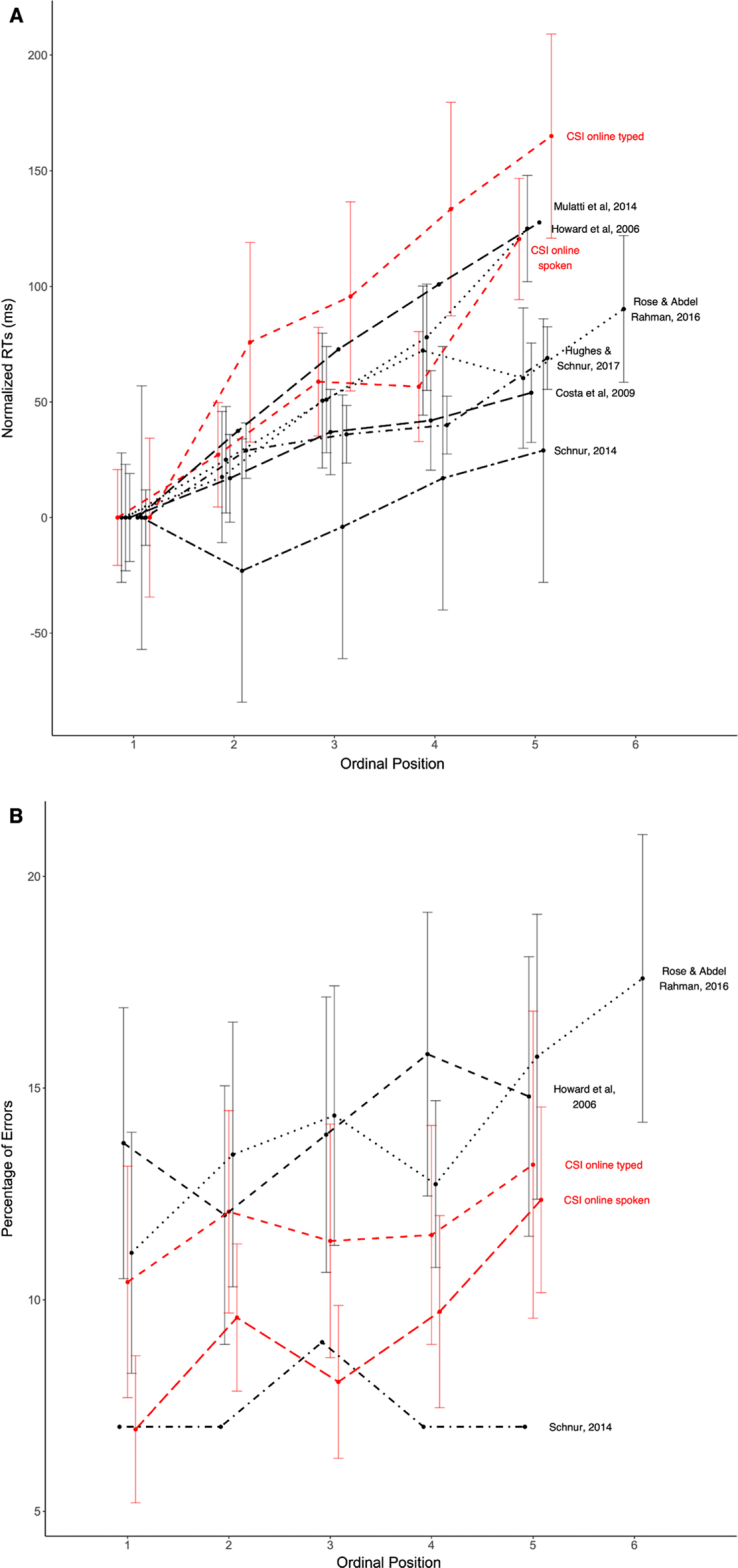
**a** Mean naming latencies (RTs) in milliseconds (normalized to the first ordinal position) and **b** mean error rates as a function of ordinal position across several experiments using the CSI paradigm. *Note*. The lab-based studies summarized here are the following: Costa et al., 2009 (only RTs); Howard et al., 2006; Hughes & Schnur, 2017 (only RTs); Mulatti et al., 2014 (only RTs); Rose & Abdel Rahman, 2016; Schnur, 2014. A detailed comparison of online and lab-based studies can be found in Table 6. Mean reaction times and error rates for each ordinal position were extracted from tables or plots reported in the respective papers or from raw data. Where available, error bars represent 95% within-subject confidence intervals (CI) around the mean. Unfortunately, the method applied for CI calculation was not always available, but most studies applied the methods suggested by Loftus and Masson (1994), Masson and Loftus (2003) or Morey (2008), whose CI sizes should be directly comparable (according to Morey, 2008). To increase the visibility of the plot, error bars were jittered around the ordinal positions. **a** To report the overall CSI effect, the mean of the first ordinal position was subtracted from the respective means of the other ordinal positions. As can be seen, the effect from spoken responses in the current online study (Experiment 1) is comparable to effects from lab-based CSI experiments, whereas typed responses (Experiment 2) resulted in a stronger cumulative semantic interference effect at a higher variance. **b** As can be seen from panel B, the means and variances of error rates from spoken and typed responses in the current study are within the range of errors in previous lab-based experiments. Taken together, both speed and accuracy of spoken responses in the current online study (Experiment 1) are comparable to lab-based CSI effects, whereas the typed responses (Experiment 2), with a comparable accuracy, result in a numerically stronger but more variable cumulative semantic interference effect (speed)

Figure 4 shows a comparison of the linear trend and error rates found in our web-based experiments with those from a collection of several lab-based experiments using the same paradigm (see Table 6 for a detailed comparison of the collection of studies). As can be seen, the magnitude of the CSI effect (RT increase per ordinal position) in our web-based experiments with spoken responses fits well into that range, whereas the typed responses yield a numerically larger CSI effect. Error rates from both experiments fit well into the range reported in the lab-based experiments.

**Table 6:** Comparison of the current web-based experiments to the collection of published laboratory-based studies taken up in Fig. 4

ID	Study	Manipulation	*N*_participants_ (gender; age)	Language	*N*_stimuli_ (*n*_*fillers*_);*N*_repetitions_	*N*_categories_ (à *n*_exemplars_); lags	Stimulus type	Max. picture presentation (ISI)	*M*_latency_ of 1^st^ ord. pos. (95% CI)	*M*_interference_ (95% CI)	Errors
	Exp. 1: Spoken naming	Online setting	30 (16 female; aged 19–35)	German	120 (+40 fillers); no repetition	24 (5); lags 2–8 (randomized)	Colour photographs	2000 ms (ca. 500 ms)	913 ms (±21)	30.8 ms (±11.5)	7.7%
	Exp. 2: Typed naming	Typewritten CSI; online setting	30 (9 female; aged 18–35)	German	120 (+40 fillers); no repetition	24 (5); lags 2–8 (randomized)	Colour photographs	6000 ms (ca. 500 ms)	1152 ms (±34)	41.7 ms (±13.4)	10.9%
1	Costa et al., 2009	ERPs and time course of CSI	24 (aged 18–25)	Spanish	120 (+40 fillers); 3 repetitions ➔ overlap: 28%	24 (5); lags 2–7 (randomized) ➔ overlap: 63%	Black and white line drawings	1500 ms (2000 ms)	809 ms (±20)	13.2 ms	8.7%
2	Howard et al., 2006	Orthogonalizing lag and ordinal position effects	24 (17 female; aged 18–38)	English	120 (+40 filler); no repetition ➔ overlap: 29%	24 (5); lags 2, 4, 6, 8 (balanced) ➔ overlap: 58%	Colour photographs	2000 ms (1250 ms)	610 ms (±23)	30.0 ms (±8.2)	22.3%
3	Hughes & Schnur, 2017 (Exp. 1)	Correlating semantic blocking, CSI, and picture–picture priming	71	English	60; no repetition ➔ overlap: 16%	12 (5); lag 2 ➔ overlap: 25%	Colour photographs	1600 ms (1000 ms)	860 ms (±12)	15.1 ms (*SD =* 13.8)	15.5%
4	Mulatti et al., 2014 (young ppt.)	CSI + repetition priming in elderly (with MCI) and young ppt.	23 (19 female; aged 19–25)	Italian	60 (+28 filler); 1 repetition ➔ overlap: 22%	12 (5); lags 2, 4, 6, 8 (balanced) ➔ overlap: 46%	NA	3000 ms (1400 ms)	700 ms	31.3 ms	8.3% (across groups)
5	Rose & Abdel Rahman, 2016 (close relation; 1^st^ repetition only)	CSI effect in close vs distant semantic relations in RTs and ERPs	24 (aged 20–39)	German	108 (+108 in distant condition + 84 filler); repetitions ➔ overlap: up to 71%*	18 (6) (+18 per ppt. in distant condition); lags 2–8 (randomized) ➔ overlap: up to 49%*	Colour photographs	2000 ms (2000 ms)	973 ms (±28)	20.0 ms (±10.1)	9.1%
6	Schnur, 2014 (Exp. 1, short RSI)	Replicate Howard et al., 2006; long vs short RSI	24	English	120 (+52 fillers); no repetition ➔ overlap: 26%	24 (5); lags 2, 4, 6, 8 (balanced) ➔ overlap: 58%	Line drawings	2000 ms (750 ms)	866 ms	14.2 ms** (±7.7)	< 12.9%**

All cited studies report a significant cumulative semantic interference by ordinal position. All relevant studies found no effect of lag (studies 2, 4, 6). Only Experiment 1 of the present study and study 5 show a statistically significant effect of ordinal position on error rates. Lags = number of intervening stimuli between exemplars of one category; ISI = inter-stimulus interval; ord. pos. = ordinal position; 95% CI = 95% within-subject confidence intervals around the mean (methods by Loftus & Masson, 1994, Masson & Loftus, 2003, or Morey, 2008 [sizes comparable according to Morey, 2008]). Errors = errors include naming errors, invalid and no-responses and microphone/voice key errors; overlap = percentage of stimuli/category (respectively) shared with the material used in the current study; ERPs = event-related potentials; MCI = mild cognitive impairment; RSI = response–stimulus interval. *up to because, across participants, all stimuli appear in close and distant semantic conditions; **across both RSIs. Missing information was not available

### Post Hoc Power Analyses

To inform future online language production experiments, we conducted post hoc power analyses for RT effects at different sample sizes and category numbers using the powerCurve function of the simr package in R (Green & MacLeod, 2016). As the function did not work for the GLMMs with gamma distributions used in our main analyses, we log-transformed the RT data and used a linear mixed model instead. Otherwise, the models were kept identical^3^ and the experimental effect sizes were used (Experiment 1: Estimate_log(RT)_ = 0.028, *p*_Satterthwaite_ < .001; Experiment 2: Estimate_log(RT)_ = 0.030, *p*_Satterthwaite <_ .001). For each estimation, the number of simulations was *n =* 1000.

**Fig. 5:**
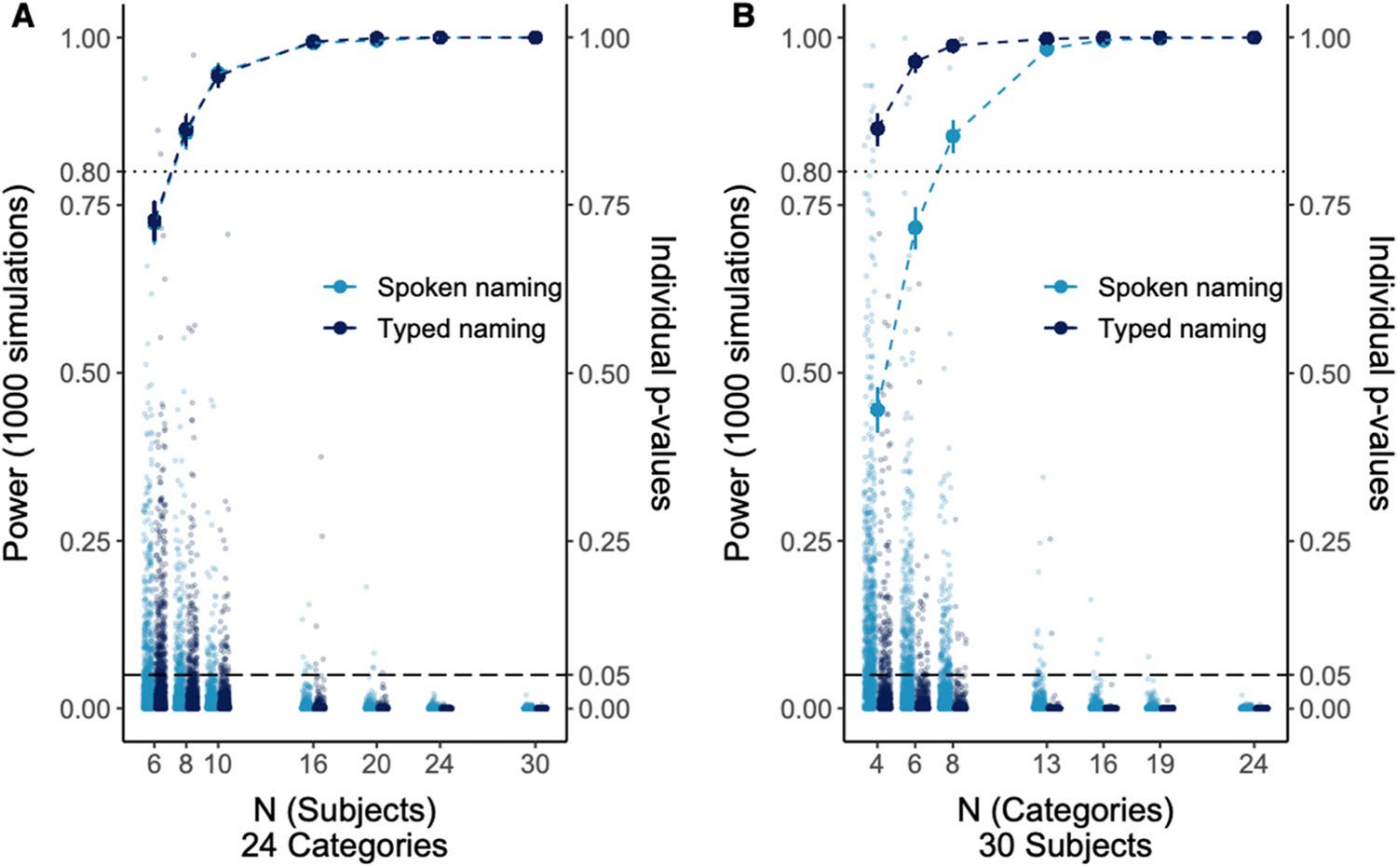
Results of the post hoc power analysis for the fixed effect of ordinal position at varying sample sizes (**a**) and category numbers (**b**) in Experiments 1 and 2. *Note.* Plots show the estimated power, i.e. the percentage of significant effects assuming that the effect is there, at a different sample sizes (and 24 categories) and b different numbers of categories (and a sample size of 30 participants). The line graphs show the estimated power for spoken (turquoise/light grey) and typed naming (dark blue/dark grey) with vertical lines representing the 95% confidence interval around the mean. The dotted horizontal line represents a power of 80%. The jittered dots represent the *p*-values for each of the 1000 simulations, and the dashed horizontal line represents a *p*-value of .05, the cut-off for a simulation to be considered significant. *X*-axis breaks were chosen such the combinations of subjects and categories resulted in similar numbers of trials in estimations displayed in plots a and b, respectively (see Table 7). Twenty-four categories and 30 participants—the break on the far right in both plots—is the actual post hoc power of the experiments

Figure 5 shows the estimated power for increasing category and sample sizes of Experiments 1 and 2. The resulting total number of trials for each of the analyses is displayed in Table 7. The power estimations at different samples sizes were almost identical for both experiments, but relatively higher at smaller category sizes for typed than for spoken responses. Both experiments (30 subjects and 24 categories × 5 exemplars) yielded significant results in each of the 1000 simulations. Power, i.e. the percentage of significant results assuming that the effect is actually there, started to decrease with 10 subjects and 24 categories or 30 subjects and eight categories (1200 trials each before trial exclusion), and dropped below 80% with six subjects in both response modalities or six categories in the spoken naming task.

**Table 7:** Numbers of trials (before trial exclusion) at different sample sizes and numbers of categories

Varying number of subjects			Varying number of categories		
*n* (Subjects)	*n* (Categories)	Total *N* trials	*n* (Subjects)	*n* (Categories)	Total *N* trials
30	24	3200	30	24	3200
24	24	2880	30	19	2850
20	24	2400	30	16	2400
16	24	1920	30	13	1950
10	24	1200	30	8	1200
8	24	960	30	6	900
6	24	720	30	4	750

## Discussion

In this study, we set out to replicate the cumulative semantic interference (CSI) effect in a web-based setting, comparing two response modalities for feasibility and validity, namely spoken and typed responses. A stable effect in lab-based language production research, the CSI effect is elicited for each new member of a previously presented category in the continuous naming paradigm with consecutive naming of seemingly unrelated pictures. In the two experiments presented here, we ran the CSI paradigm through participants’ web browsers using the platform SoSci Survey. In Experiment 1, the participants’ microphones were accessed, and their *spoken* responses were recorded through a JavaScript implementation. In Experiment 2, the first keystroke of each *typed* target word was used as the response variable, which was assessed by a custom JavaScript plugin (Stark, 2021b). In both online experiments, we were able to replicate the linear increase in reaction times for each additional category member. Additional exploratory analyses showed that error rates also increased for additional category members in spoken, but not in typed responses. Although a direct comparison between lab-based and online assessment was not performed, we show that, overall, both speed and accuracy matched well with previous lab-based studies. Our results thereby add to the growing body of evidence that language production research can be conducted in online settings (Fairs & Strijkers, 2021; Vogt et al., 2021). Moreover, we show that measures of typewritten responses provide a valuable tool for online language production research which can be automatically analysed, thus reducing workload and time investment for data processing.

### Comparison between different response modalities (spoken/typewritten)

The results of Experiment 1 using overt spoken responses revealed a stable CSI effect with a linear trend of ~31 ms. Moreover, an analysis of overall errors revealed a significant increase in error rates across ordinal positions. The effect is quite large, which may be related to the use of semantic subcategories with closely related items. Such narrow categories have been shown to result in stronger CSI effects compared to main categories with distantly related items (Rose & Abdel Rahman, 2016). Moreover, the randomization in our design was done within blocks of categories, rather than across the whole stimulus list, leading to a slight predominance of short compared to long lags between category members, which may additionally have increased the effect, although previous research suggests that lag does not strongly affect the linear increase of reaction times in the CSI paradigm (e.g. Schnur, 2014).

In Experiment 2, we assessed typing as an alternative response modality to measure reaction times in language production research. Both handwritten and typewritten responses have previously been used in picture-naming experiments (Baus et al., 2013; Bonin et al., 2002; Pinet et al., 2015; Pinet, Dubarry, & Alario, 2016a; Qu et al., 2016; Qu & Damian, 2020; Torrance et al., 2018; Zhang & Damian, 2010). However, to the best of our knowledge, no study using typewritten responses has tested semantic interference effects. Our study is therefore the first to provide evidence on this response modality in a reaction time-dependent semantic interference task such as the CSI paradigm. We find a strong and stable CSI effect also for typed responses. This effect of ~42 ms per ordinal position is numerically even stronger when compared to Experiment 1 (spoken responses) and to comparable lab-based CSI experiments. Moreover, the effect shows a larger variance, and typed responses were overall much slower than spoken responses (by ~100 ms). The latter is in line with previous reports (e.g. Bonin & Fayol, 2000). Additionally, latency also increased with ordinal position. Additionally, latency differences in the present study may stem from the different stimulus familiarization procedures in Experiments 1 and 2. In the spoken naming task, participants saw the pictures in groups of eight pictures each and proceeded in a self-paced manner, while in the typewritten naming task, participants were familiarized with the pictures individually and typed each picture name. Research suggests that the overt production of the picture names may lead to deeper processing of both the visual details and the verbal labels of pictures (Hourihan & Churchill, 2020), which in turn can affect naming latencies. Yet another explanation of the longer naming latencies could be a technical one. Comparing actual keystroke latencies and the latencies recorded online, previous studies reported online latencies for each keypress to be ~30–100 ms slower than the actual latencies, depending on the hardware, operator system and browser used (Pinet et al., 2017; Reimers & Stewart, 2015). While it seems unlikely that the ~100 ms overall difference of spoken and typewritten latencies reported here can be explained uniquely by a smaller technical delay in the audio recordings, an actual comparison of lab-based and online recorded latencies is pending. Beyond these technical caveats, the robust demonstration of the CSI effect for the typed modality suggests an origin at the lexico-semantic processing level independent of output modality, as proposed by most theoretical accounts of cumulative interference (e.g. Levelt et al., 1999; Roelofs, 2018). Although not a primary target of the present study, similar CSI effects between the two modalities speak for an origin at the conceptual or lexical level (Abdel Rahman & Melinger, 2009, 2019; Howard et al., 2006; Oppenheim et al., 2010). An origin at the articulatory or word form level, as has been proposed based on picture-word interference tasks (Mahon et al., 2007; Navarrete et al., 2010), would predict substantial differences between the two modalities tested here.

While latency effects dominate in neurotypical participants, semantic interference has also been reported in increased error rates, especially in the PWI or blocked cyclic naming paradigm (e.g. Belke et al., 2005; Caramazza & Costa, 2000; Damian et al., 2001; Gauvin et al., 2018; Starreveld & La Heij, 2017). In the CSI task, the effect of semantic interference on error rates is still inconclusive as only some studies did find error effects by ordinal position (Howard et al., 2006; Schnur, 2014 vs Rose & Abdel Rahman, 2016). This may be one reason why we found an error-based CSI-effect for the spoken, but not in the typed responses. The longer overall latency for typed responses and the different quality of the potential sources of errors (e.g. keystroke accuracy due to motoric/typing skills) may have obscured the effect for typed responses in the present study. The cumulative interference in error rates for the spoken response modality, however, aligns with models assuming a lexico-semantic locus of the effect, where inhibited target retrieval may result in slower naming latencies as well as erroneous naming (Abdel Rahman & Melinger, 2009, 2019; Levelt et al., 1999; Oppenheim et al., 2010; Roelofs, 2018; Schnur, 2014).

Taken together, these data supplement previous studies showing that effects that are already well established in spoken naming can also be found in written naming (Pinet & Nozari, 2018; Torrance et al., 2018). This highlights that a number of the experimentally described effects are related to linguistic processes which support language production supra-modally. Our study shows that this holds for the CSI effect which most plausibly arises at a level independent of the output modality. The finding is encouraging for other aspects of language production research. Notwithstanding, we may highlight that in instances which require modality-specific processing, the difference is expected to be relevant. This pertains, for instance, to the assessment of articulation-related processes, or research on written language processing per se. Moreover, in elderly participants, typed (as opposed to handwritten) responses may not be as fluent, an aspect which is also of great importance when including participants with an acquired brain lesion.

## Methodological implications: Lab-based CSI effects can be replicated online

Our findings support the feasibility of collecting overt language production samples from participants at their homes using JavaScript-based plugins which can be implemented in many online platforms. This could be particularly useful to collect data from participants across different time points, nationalities or social backgrounds, increasing the diversity of the sample usually included in psycholinguistic research.

It also opens the perspective to test participants with an acquired language disorder (most notably stroke-induced aphasia). Long-term follow-up, especially regarding scientifically motivated questions, is often hampered by the efforts related to re-inviting and transporting the patient to the respective institution. As a caveat, computer competence and access to web browsers need to be assessed in such populations. Moreover, distortions of articulation (spoken modality) and/or impairments of fine motor skills (typing) need to be respected. The fact that we showed qualitatively similar effects for both modalities is encouraging, potentially allowing for the use of the respectively less impaired modality.

In most experiments investigating keystroke latencies or typewritten responses, participants were screened for their typing abilities, restricting the analyses on skilled or expert typists (Pinet et al., 2015; Pinet, Dubarry, & Alario, 2016a; Scaltritti et al., 2017; but see also e.g. Baus et al., 2013). Our results show that refraining from such restrictions still allows for the robust demonstration of a semantic interference effect. By including the “normal” range of typing abilities, we were able to collect our participants from the same population as in Experiment 1. Furthermore, the fact that the CSI effect can be found across a relatively wide range of typing abilities suggests its high reliability even in online settings. However, it should be noted that people subscribed to online experimental platforms such as Prolific are probably more experienced typists, an issue which will be of relevance in elderly populations and in people with an acquired language or cognitive and/or motor deficit. We will address this issue in a follow-up study including participants with mild to moderate aphasia.

## Technical implications: Reducing preprocessing efforts

In a previous language production experiment using SoSci Survey and the JavaScript plugin described here (Vogt et al., 2021), the file lengths of the sound recordings were reported to vary between or even within participants, the reason for this variation being still unknown (for a discussion on possible reasons see below). This was the case also in the current sample. We therefore additionally assessed the interaction of ordinal position and file length (*z*-transformed) in a statistical model. The interaction term was not significant (estimate = 4.37, *SE* = 3.8, *t* = 1.147, *p* = .251), meaning that variation in file lengths did not influence the effect.

Importantly, our results are encouraging regarding an aspect of preprocessing of the data. Since both options (spoken vs typed responses) yielded similar effects, the cumbersome preprocessing of spoken responses may be eased by the use of typewritten responses in some research scenarios. Despite automated vocal onset detection through algorithms such as that provided by Chronset (Roux et al., 2017), all data has to be double-checked by the (native speaker) experimenter for accuracy of the response and the VOT, resulting in potentially hours or days worth of workload. This may be especially challenging if the data quality is poorer in online when compared to the lab-based acquisition (Fairs & Strijkers, 2021; Vogt et al., 2021), increasing the need to carefully check the data.

Within the typewritten response modality, we were able to drastically reduce data-processing efforts. With a custom R script (Stark, 2021a) and an R package with functions for comparing string inputs (van der Loo, 2014), we tested automated classification of the typed responses. As this method produced near-identical classification when compared to manual processing, and an identical statistical effect, it is an effective way to reduce workload in language production experiments. Beyond doubt, spoken production is the most relevant target. However, to make large cohort assessments possible, the typewritten response modality may complement a number of exciting research questions to be addressed in the field.

## Recommendations for running language production experiments online

Based on the two experiments reported here, we may highlight some recommendations for future experiments. For both the spoken and typewritten modality, we observed large effects at high power. While the power remains high even for a relatively small number of trials, our post hoc power analysis for the spoken response modality suggests that the number of trials per participant, i.e. the number of categories in the CSI task, affects the power more strongly than the number of participants, confirming previous reports (Vogt et al., 2021). Thus, a reasonable number of trials per participant should be implemented. Paradigms with many trials and within-participant manipulations such as the CSI paradigm reported here seem to elicit robust effects, potentially counteracting the negative effects of a less controlled setting at the participants’ homes compared to a lab environment, technical disturbances or potential non-compliance. This may allow for testing more diverse populations to increase ecological validity.

For the technical implementation of both audio recordings and typewritten latencies, we recommend lean JavaScript-based implementations. JavaScript-based plugins such as the ones used and presented here are a good alternative that give researchers full control over the script. Recently, some platforms for online experiments have started to implement audio recordings already into their predefined tools and functions (e.g. Gorilla Experiment Builder, Anwyl-Irvine, Massonnié, et al., 2020b or FindingFive, FindingFive Team, 2019). The assessment of keystroke latencies is an inbuilt feature of most JavaScript-based platforms. We have not yet tested these inbuilt features, but assume that they should lead to very similar results as the custom scripts. Predefined tools and functions may thus be a good alternative for researchers who prefer easy-to-handle implementations, including drag-and-drop programming, rather than customizing code. Independently of the implementation used, it is important to note that from the current study, we cannot draw assumptions on the actual degree of systematic bias or technical noise introduced by different hardware/software set-ups. With our fully randomized within-subject design, we were able to replicate hypothesized effects at high power despite potential noise; however, this cannot be transferred to pseudo-randomized and/or between-subject designs.

In the spoken naming task, like other authors (Vogt et al., 2021), we observed some variation in audio recording file lengths. This variation did not affect the effect reported here. Still, we do not yet know the source of the variation, and it may have occurred at the beginning or at the end of the recording. Therefore, researchers should pay particular attention to this potential source of noise. Crucially, only a variation at the beginning of the recordings should affect the assessment of reaction times. A simple method to improve the synchronization of the audio recording (timer for typewritten answers) and stimulus presentation is to present the stimulus and to start the recording (or timer) only after the page is fully loaded. This can be achieved by using the *window.onload* event in JavaScript. Although it may lead to some jittering of the interstimulus interval (depending on the internet connection), like this, the stimulus can be preloaded in every trial, leading to a high synchronization of reaction time measurements (audio or typed) and stimulus presentation. We therefore recommend all researchers to make use of such a method.

For typewritten answers, we compared manual and automatic processing procedures and found they classified nearly all typewritten answers identically as correct or incorrect. We therefore highly recommend such automatic classification procedures. However, researchers should decide a priori which classification procedure to use and which cut-off criterion to apply. Different procedures exist that are specialized for different typewritten answers, such as longer texts and or single words. We recommend Bosker (2021) and van der Loo (2014) as overview articles. We found that the *Jaro distanc*e (Jaro, 1989, 1995), a method specialized for short answers, led to good results with a cut-off criterion of *d =* .3. The even more widely known *Levenshtein distance* (Levenshtein, 1966; all operations equally weighted; cut-off criterion *d* = .3) and the *optimal string alignment* procedure (restricted Damerau Levenshtein distance; all operations equally weighted; cut-off criterion *d =* .3) as implemented in the stringdist package also led to very good, though slightly more conservative, results (see Appendix 3). Furthermore, researchers must provide the algorithms with accepted naming alternatives (e.g. “sofa” instead of “couch”). In the best case, these lists can be compiled based on previous experiments. If no such prior data exists, researchers should carefully check their data after the classification. Beyond such caveats, using automated classification procedures not only reduces the time needed for data preprocessing from hours to seconds (Borrie et al., 2019), it also increases the inter-rater reliability. For a follow-up experiment, we even implemented these simple methods into the experiment itself, in order to provide feedback on the typing accuracy already during the experiment. Before running an experiment with typewritten responses, researchers must decide whether participants’ typed answers should be displayed on the screen and whether participants are allowed to correct their typewritten answers by using the backspace key. Displaying participants' answers on the screen can both affect naming latencies (Perret & Laganaro, 2013; Snyder et al., 2015) and error types (Pinet & Nozari, 2020, 2021). However, in online settings, we assume that giving no feedback at all may reduce adherence to the task. Anecdotal evidence suggests that allowing corrections in typewritten answers is more similar to natural typing behaviour, but it may result in different overall typing duration. If, like in our case, researchers are most interested in typing onset times, they may decide to allow corrections in typewritten answers. If, by contrast, researchers are interested in inter-keystroke intervals and overall typing duration, they may decide not to allow corrections.

Last but not least, we recommend restricting the use of different keyboard types. Often, one keyboard type has direct key bindings for all letters of a language, while others do not. This can affect the number of keys that need to be pressed to type a specific word and the motor preparation stages if the same letter is to be pressed with different hands on different keyboards (Pinet, Dubarry, & Alario, 2016a). Together with our script to assess typing latencies, we provide one example how the keyboard type can be assessed. Next to restricting the keyboard type, at least for languages including accents or capitalization at the beginning of a word, we recommend instructing participants to use the caps lock key and to write all letters in upper case.

## Conclusion

Running experiments online opens new perspectives for assessing more diverse populations across different linguistic, social or generational backgrounds. Our study adds evidence to the feasibility of implementing reaction-time-sensitive language production experiments in web-based settings. This allows for running cross-linguistic, cross-sectional or longitudinal studies which may have limited practicability in in-person, lab-based settings. Moreover, we show that typewritten responses are a valid, practical alternative to collecting overt spoken responses through participants’ microphones. Automatic processing can further reduce the workload of processing the typewritten answers. By highlighting important technical and conceptual considerations, we hope to have provided recommendations for an easy access to studying both typewritten and spoken language production online.

## Figures and Tables

**Fig. 6:**
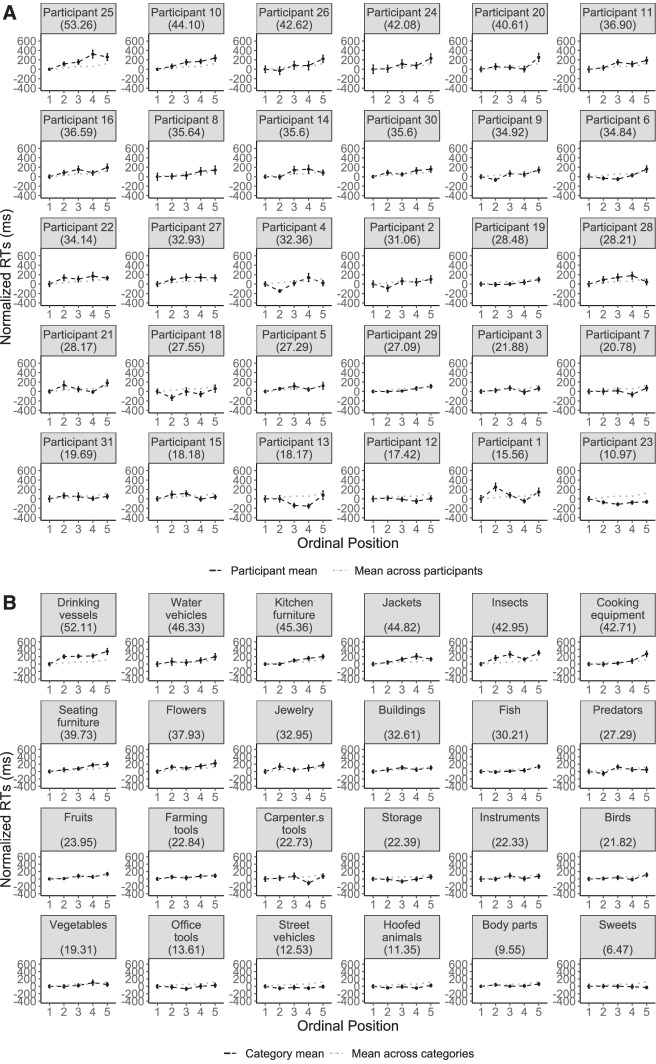
Normalized mean naming latencies (RTs) in milliseconds as a function of ordinal position for each (**a**) participant and (**b**) category in Experiment 1. *Note.* Mean normalized reaction times in milliseconds per ordinal position. Subplots show the reaction times for each participant (category) separately, ordered by the size of their CSI effect, i.e., the average increase per ordinal position as estimated by the GLMM (see results section) shown in grey boxes above each graph. Mean reaction times were calculated across semantic categories (participants). The RTs were normalized for each participant (category) by subtracting the mean of the first ordinal position from the respective means of the other ordinal positions. The grey dotted line shows the mean RT per ordinal position across participants and categories. Error bars show standard errors of the mean. Values were adjusted for within-participant designs using the method suggested by Morey (2008) as implemented in the *summarySEwithin( )* function from the R package Rmisc (Hope, 2013)

**Fig. 7:**
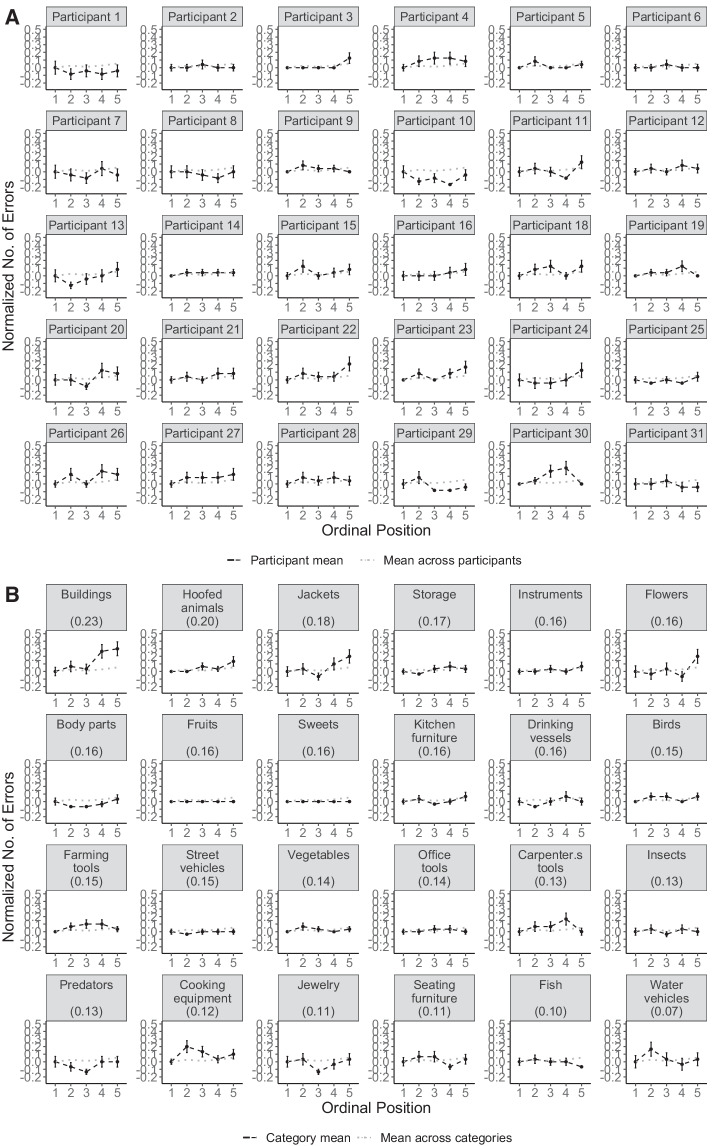
Normalized error rate in percent as a function of ordinal position for each (**a**) participant and (**b**) category in Experiment 1. *Note.* Mean normalized error rates per ordinal position. Subplots show error rates for each participant (category) separately. Mean error rates were calculated across semantic categories (participants). The error rates were normalized for each participant (category) by subtracting the mean of the first ordinal position from the respective means of the other ordinal positions. The grey dotted line shows the mean error rate per ordinal position across participants and categories. Error bars show standard errors of the mean. Values were adjusted for within-participant designs using the method suggested by Morey (2008) as implemented in the *summarySEwithin( )* function from the R package Rmisc (Hope, 2013)

**Fig. 8:**
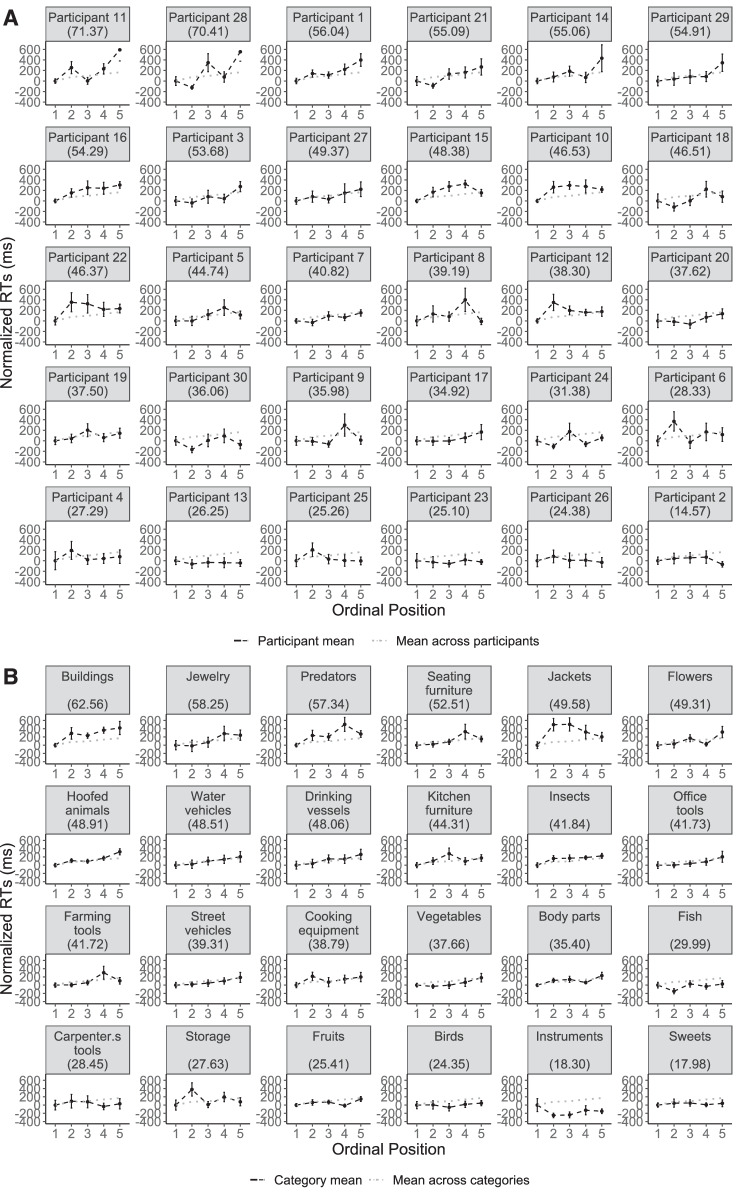
Normalized mean typing latencies (RTs) in milliseconds as a function of ordinal position for each (**a**) participant and (**b**) category in Experiment 2. *Note.* Mean normalized reaction times in milliseconds per ordinal position. Subplots show the reaction times for each participant (category) separately, ordered by the size of their CSI effect, i.e., the average increase per ordinal position as estimated by the GLMM (see results section) shown in grey boxes above each graph. Mean reaction times were calculated across semantic categories (participants). The RTs were normalized for each participant (category) by subtracting the mean of the first ordinal position from the respective means of the other ordinal positions. The grey dotted line shows the mean RT per ordinal position across participants and categories. Error bars show standard errors of the mean. Values were adjusted for within-participant designs using the method suggested by Morey (2008) as implemented in the *summarySEwithin( )* function from the R package Rmisc (Hope, 2013)

**Fig. 9:**
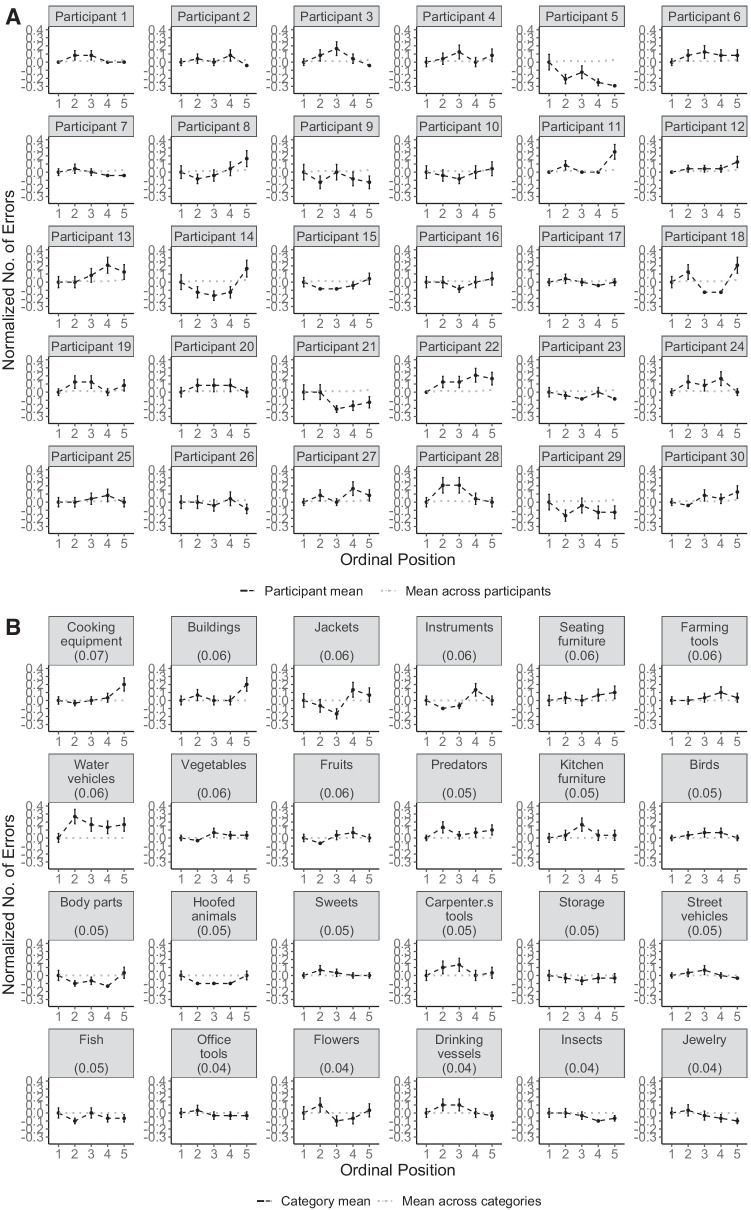
Normalized error rate in percent as a function of ordinal position for each (**a**) participant and (**b**) category in Experiment 2. *Note.* Mean normalized error rates per ordinal position. Subplots show error rates for each participant (category) separately. Mean error rates were calculated across semantic categories (participants). The error rates were normalized for each participant (category) by subtracting the mean of the first ordinal position from the respective means of the other ordinal positions. The grey dotted line shows the mean error rate per ordinal position across participants and categories. Error bars show standard errors of the mean. Values were adjusted for within-participant designs using the method suggested by Morey (2008) as implemented in the *summarySEwithin( )* function from the R package Rmisc (Hope, 2013)

## Appendix 1: List of Stimuli

**Table 8:** Experimental stimuli (English translations in brackets) and acceptable synonyms ordered alphabetically by semantic subcategories

**Items**	**Acceptable alternatives**	**Items**	**Acceptable alternatives**
**Birds**		**Farming tool**s	
Ente (duck)		Axt (axe)	Beil
Eule (owl)	Uhu	Besen (broom)	Strohbesen
Schwan (swan)		Säge (saw)	Handsäge
Strauss (ostrich)	Pfau, Strauch	Schaufel (shovel)	Spaten, Schippe
Taube (pidgeon)		Sense (scythe)	Sichel
**Body parts**		**Flowers**	
Arm (arm)		Löwenzahn (dandelion)	
Bein (leg)		Orchidee (orchid)	
Fuss (foot)		Rose (rose)	
Hand (hand)		Sonnenblume (sunflower)	
Ohr (earring)		Tulpe (tulip)	
**Buildings**		**Fruits**	
Burg (fortress)	Schloss	Apfel (apple)	
Hochhaus (skyscraper)	Wolkenkratzer	Banane (banana)	
Kirche (church)		Birne (pear)	
Schloss (castle)	Palast, Burg	Kirsche (cherry)	
Tempel (temple)	Pantheon, Ruine, Akropolis	Trauben (grapes)	Weintrauben
**Carpenter's tools**		**Hoofed animals**	
Bohrmaschine (drill)	Bohrer, Akkuschrauber	Kamel (camel)	
Feile (rasp)	Reibe, Spachtel, Hobel	Kuh (cow)	
Hammer (hammer)		Pferd (horse)	
Schraubenzieher (screwdriver)	Schraubenschlüssel, Schraubendreher	Reh (dear)	
Zange (pliers)		Schaf (sheep)	Lamm
**Cooking equipment**		**Insects**	
Gabel (fork)		Ameise (ant)	
Kelle (ladle)	Suppenkelle, Rührkelle	Biene (bee)	Bienchen
Löffel (spoon)		Fliege (house fly)	Mücke
Messer (knife)		Marienkäfer (ladybird)	Käfer
Schneebesen (whisk)	Quirl, Rührbesen, Mixer	Spinne (spider)	
**Drinking vessels**		**Instruments**	
Becher (plastic cup)	Knobelbecher, Pappbecher	Geige (violin)	Violine
Flasche (bottle)	Glasflasche, Wasserflasche	Gitarre (guitar)	
Glas (glass)	Becher	Harfe (harp)	
Kanne (teapot)	Teekanne, Teekessel, Kaffeekanne	Klavier (piano)	Piano
Tasse (tea cup)	Teetasse, Becher	Schlagzeug (drum kit)	
**Jackets**		**Seating furniture**	
Arztkittel (lab coat)	Chemiekittel, Kittel, Laborkittel	Bank (bank)	
Daunenweste (down vest)	Weste	Couch (couch)	Sofa, Ledercouch
Kapuzenpulli (sweater)	Pulli, Pullover, Hoodie, Kapuzenpullover	Hocker (stool)	Schemel
Pelzmantel (fur coat)	Mantel, Pelz, Fellmantel	Sessel (armchair)	
Sakko (sport coat)	Anzug, Jackett	Stuhl (chair)	
**Fish**		**Storage**	
Aal (eel)		Kleiderschrank (wardrobe)	Schrank
Delfin (dolfin)		Regal (cupboard)	
Goldfisch (goldfish)	Fisch	Safe (safe)	Tresor
Hai (shark)	Haifisch	Schachtel (box)	Kiste, Box, Kasten, Schachtel, Karton, Schuhbox
Rochen (ray)	Mantarochen, Manta	Schublade (drawer)	Schubkaste
**Jewelry**		**Street vehicles**	
Armband (bracelet)	Armband, Armkette, Armreif	Auto (car)	SUV, Mercedes
Brosche (brooch)	Schmuck, Amulett, Diadem, Juwelen, Schmuck, Haarspange	Bus (bus)	
Kette (necklace)	Collier, Halskette, Perlenkette, Halsband	Kutsche (carriage)	
Ohrring (earring)	Ohranhänger	Lkw (truck)	Lastkraftwagen, Laster, Lastwagen, Transporter
Ring (ring)	Goldring, Ehering	Motorrad (motorcycle)	
**Kitchen furniture**		**Sweets**	
Geschirrspüler (dishwasher)	Geschirrspülmaschine, Spülmaschine, Geschirrwaschmaschine, Spüler	Bonbon (candy)	
Herd (stove)	Kochfeld, Herdplatte, Ofen, Ofenplatte	Eis (ice cream)	Schokoladeneis, Eiscreme
Kaffeemaschine (coffee machine)	Kaffeekocher	Kekse (biscuit)	Keks, Schokoladenkeks, Cookies
Kühlschrank (fridge)	Gefrierschrank	Kuchen (cake)	Torte
Mikrowelle (microwave)		Schokolade (chocolate)	
**Office tools**		**Vegetables**	
Bleistift (pencil)	Stift	Brokkoli (broccoli)	
Klammer (paper-clip)	Büroklammer, Clip, Briefklammer	Gurke (cucumber)	Salatgurke
Lineal (ruler)	Zollstock	Karotte (carrot)	Möhre
Radiergummi (rubber)	Radierer	Kartoffel (potato)	
Schere (scissors)		Paprika (bell pepper)	Grüne Paprika
**Predatory animals**		**Water vehicles**	
Bär (bear)	Braunbär	Gondel (gondola)	Gondola, Gondelier, Boot
Leopard (leopard)	Gepard, Jaguar, Puma	Kanu (canoe)	Boot
Löwe (lion)		Segelschiff (sailing boat)	Modellschiff, Segelboot, Schiff, Boot
Panther (panther)	Puma, Jaguar, Gepard	U-Boot (submarine)	Unterseeboot, Militärschiff, Boot
Tiger (tiger)		Yacht (yacht)	Schiff, Boot

## Appendix 3: Experiment 2–Comparison of Further Automated String Matching Procedures

To date, there are a wide range of string matching metrics available, each with specific characteristics and applications. In our main analyses, we chose to use the Jaro distance (cut-off: *d*_Jaro_ ≥ .3; Jaro, 1989, 1995) because this metric was tailored specifically to short string inputs. Here, we compare the manual and automated classifications with further string matching metrics, the Jaro-Winkler distance (*p =* 0.1; cut-off: *d* ≥ .3), the Levenshtein distance (equal weights of 1 each; cut-off: *d* ≥ 3), the optimal string alignment (also called restricted Damerau-Levenshtein distance; equal weights of 1 each; cut-off: *d* ≥ 4), and the Jaccard bi-gram distance (*q =* 2; cut-off: *d* ≥ 0.8). For all metrics, we used our custom preprocessing functions (see description in the methods section of this manuscript; https://github.com/kirstenstark/stringmatch_typed_naming), and the technical implementations from the *stringdist* package (version 0.9.6.3; van der Loo, 2014)*.* For the exact formula applied, we may refer to van der Loo (2014).

As shown in Appendix Table 9, all metrics yielded very similar classifications that were close to perfectly correlated with our intuitive, manual classification. The numerically highest correlation was found between the manual classification and the Levenshtein distance and the optimal string alignment metric. Those were also somewhat more conservative, because, in comparison to the other metrics, they classified slightly less words as correct that intuitively were classified as incorrect and more words as incorrect that intuitively were classified as correct.

**Table 9:** Comparison of different automated string matching metrics

Metric	*r* (manual)	“New correct” words			“New incorrect” words			
		Partial name	Orthograph. similarity	Loosely related	1^st^ letter backspace	Phonolog. similarity (1^st^ letter)	Distance-based	Other
Jaro	.969	5	2	1	13	6	1	1
Jaro-Winkler	.962	*5*	2	6	1﻿4	6	1	-
Levenshtein	.971	*-*	-	1	14	6	6	-
Optimal string alignment	.971	*-*	1	1	14	6	5	-
Bi-gram (Jaccard)	.963	4	2	1	15	6	4	2

*r* (manual) = Pearson’s *r* correlation of the manual classification and the respective string matching metric; “New correct” words = Number of typed words manually classified as incorrect, but as correct with the respective metric, leading to lower trial exclusion; “New incorrect” words = Number of typed words manually classified as correct, but as incorrect with the respective metric, leading to higher trial exclusion; Partial name = when participants typed only parts of the picture name (e.g. GESCHIRR [engl. *dish*] instead of GESCHIRRSPÜLER [engl. *dishwasher*]); Orthograph. similarity = when participants typed orthographically similar words (e.g. KESSEL [engl. *kettle*] instead of KELLE [engl. *ladle*]); Loosely related = when participants typed words that were semantically related to the target word, but no accepted alternative (e.g. SCHEMEL [engl. taboret] instead of STUHL [engl. chair]); 1^st^ letter backspace = when participants backspace-corrected an accepted alternative, changing the first character of the word entry (e.g. BURBackspaceBackspaceBackspaceBackspaceSCHLOSS [engl. *castl(e)…fortress*]); Phonolog. similarity (1^st^ letter) = when participants misspelled the beginning of a word with a phonologically similar phoneme (e.g. PFEILE instead of FEILE [engl. similar to *wrasp* instead of *rasp*]), Distance-based = when the computed distance was higher than the respective cut-off; Other = classification differences that were difficult to classify

## Appendix 4: Experiment 2–Analyses of Manually Classified Typewritten Answers

Here, we report the results of the preregistered RT data analysis of the manually/half-automatically classified data from Experiment 2. For a description of the procedures, we may refer to the methods section of this manuscript.

The mean reaction times, i.e., the latencies between picture onset and first keystroke show a linear increase with ordinal position (Appendix Table 10). The GLMM confirmed this linear trend: As with the automatically pre-processed data, RTs increased significantly with an average of ~ 42 ms per additional member of each category (Appendix Table 11).

**Table 10:** Typing latencies in milliseconds (RTs) and erroneous trials for each ordinal position

Typing latencies (RTs)	Ordinal position				
	1	2	3	4	5
*M*	1151.61	1224.81	1247.35	1285.51	1317.46
*SEM*	17.43	21.77	20.67	23.53	22.44
Erroneous trials	73	84	79	83	94

*M =* mean; *SEM =* standard error of the mean; Erroneous trials *=* Number of trials per ordinal position that were excluded due to errors (technical or answer-based). *SEMs* were adjusted for within-participant designs using the method suggested by Morey (2008) as implemented in the *summarySEwithin( )* function from the R package Rmisc (Hope, 2013)

**Table 11:** Generalized Linear Mixed Model (GLMM) with gamma identity link function predicting typing latencies (RTs) by ordinal position

Effect	Estimate	*SE*	95% CI		*t*-value	*p*
			*LL*	*UL*		
Model: RT ~ ordinal position + (ordinal position | subject) + (ordinal position | category)						
Fixed effects						
Intercept	1296.05	12.26	1272.01	1320.09	105.68	**<0.001**
Ordinal position	42.32	7.21	28.18	56.46	5.87	**<0.001**

Number of participants = 30; number of categories = 24; total *N =* 3187; *SE =* standard error, *CI =* confidence interval around the estimate, *LL = *lower limit, *UL =* upper limit. Significant *p*-values of* p* < .05 are shown in bold

## Footnotes

1 One of these participants was excluded based on her comment that she was living abroad and was no longer used to typing in German. The other two were excluded due to trial loss >20%.

2 To date, there are several distance metrics available, each with specific characteristics and applications. We chose to base our analysis on the Jaro distance because this metric was tailored specifically to short string inputs. A comparison between different string matching metrics, including the Jaro–Winkler distance, the Levenshtein distance, the restricted Damerau–Levenshtein distance and the Jaccard bi-gram distance, is reported in Appendix 3.

3 Only the model for typewritten responses was slightly adjusted. It converged only after the number of iterations was increased to 2 × 105 and the random slope of the ordinal position for the categories was dropped.
